# Protecting the invisible health workforce: experiences and occupational safety challenges of facial protective equipment use among central sterile supply department workers

**DOI:** 10.3389/fpubh.2026.1944828

**Published:** 2026-10-14

**Authors:** Huiling Rao, Aliya Tuerdi, Xue Zhou, Xiaomei Ren

**Affiliations:** Department of Medical Engineering, The First Affiliated Hospital (Southwest Hospital) of Army Medical University, Chongqing, China

**Keywords:** adherence, central sterile supply department, facial personal protective equipment, healthcare workers, occupational protection, qualitative research

## Abstract

**Background:**

Central sterile supply department (CSSD) personnel may be exposed to blood, body fluids, splashes, and aerosols while reprocessing contaminated medical devices. Evidence remains limited on how CSSD personnel experience facial personal protective equipment (facial PPE) during routine work and what shapes their reported protective practices. This study explored CSSD personnel’s experiences of facial PPE use and the factors they perceived as influencing these practices.

**Methods:**

A qualitative descriptive study was conducted from January to February 2026 in the CSSD of a tertiary hospital in Chongqing, China. Purposive sampling was used to recruit frontline personnel who had worked in the CSSD for at least 1 year and routinely used facial PPE. Data collection ceased after two consecutive interviews produced no new themes. Individual semi-structured interviews lasting 30–45 min were audio-recorded and transcribed. Data were analyzed using an inductive thematic approach with NVivo 12. Two researchers independently reviewed and coded the transcripts, compared their interpretations, and developed and refined the themes through discussion.

**Results:**

Twenty CSSD personnel participated in the study. Six themes were identified: ① perceptions of facial protection as a means of safeguarding personnel, medical devices, patients, and others; ② protective decisions shaped by perceived risk, work context, comfort, and emergency conditions; ③ policies, multilevel training, supervision, and peer feedback supporting standardized facial protection; ④ discomfort, poor fit, fogging, and communication difficulties associated with facial PPE; ⑤ the influence of heat, inadequate ventilation, repetitive workflow, and prolonged continuous work on sustained use; and ⑥ differences in risk perception, workplace relationships, and passive responses to mandatory requirements. Participants recognized the value of facial protection, but reported facial PPE practices were shaped by the interaction between equipment characteristics, physical working conditions, organizational arrangements, and interpersonal dynamics.

**Conclusion:**

Participants’ reported facial PPE practices reflected the interaction of perceived risk, equipment usability, working conditions, workflow, institutional support, and workplace relationships. The findings suggest that task-based protective requirements, well-fitting and tolerable equipment, practical training, supportive feedback, and environmental and workflow adjustments may support appropriate and sustained facial PPE use in CSSDs.

## 1 Introduction

Central sterile supply departments (CSSDs) are responsible for the decontamination, inspection, assembly, packaging, sterilization, storage, and distribution of reusable medical devices (1). CSSD personnel usually work outside direct patient-care settings, yet the safety and reliability of their work affect the broader clinical care pathway. Inadequately reprocessed instruments may compromise infection prevention and patient safety, while disruption of sterile processing services may delay surgical and other invasive procedures (2). CSSD personnel constitute a safety-critical but comparatively under-recognized segment of the health workforce.

Occupational exposure is embedded in routine CSSD work. Personnel in decontamination areas handle instruments contaminated with blood, body fluids, and other organic material and may encounter splashes or aerosols during manual cleaning, flushing, and the processing of complex or lumened devices (31). They may also be exposed to sharps, detergents, disinfectants, heat, humidity, and equipment noise. Facial personal protective equipment (facial PPE), including medical masks, goggles, and face shields, is used to reduce exposure of the eyes, nose, mouth, and facial skin (3). Correct and sustained use is an important component of occupational protection in CSSDs.

Existing research indicates that healthcare workers’ adherence to PPE requirements is influenced by more than protective knowledge alone. Access to appropriate equipment, fit and comfort, practical training, perceived risk, workload, time pressure, managerial support, and workplace safety culture may all affect whether PPE is used consistently and correctly (4, 5). Facial PPE may also cause discomfort, fogging, impaired communication, or interference with task performance during prolonged use (6, 7). These findings place PPE use within the wider conditions of work rather than treating it solely as an individual behavioral choice.

Most of the current evidence was generated during infectious disease emergencies, particularly the COVID-19 pandemic (8, 9). Protective practices in those settings were shaped by rapidly changing guidance, equipment shortages, heightened risk perception, and crisis working conditions (9). The study populations were also dominated by physicians, nurses, and other healthcare workers involved in direct patient care. Such evidence may not fully explain PPE use during routine hospital operations or among personnel whose occupational exposure arises from contaminated medical devices, technical procedures, and the physical work environment.

In CSSDs, facial protection must be maintained while personnel perform tasks requiring visual inspection, manual dexterity, communication, coordination, and timely instrument processing (10, 11). The work is repetitive, technically demanding, and closely tied to the continuity of clinical services (12). Published evidence has paid limited attention to how CSSD personnel experience facial PPE during routine work or how equipment characteristics, perceived risk, task demands, environmental conditions, work organization, and institutional support shape its sustained and correct use.

This qualitative descriptive study explored CSSD personnel’s experiences of using facial PPE during routine work and the factors shaping their reported protective practices. Particular attention was given to how personnel described their protective practices across different work areas, tasks, and everyday working conditions. The study aimed to provide a detailed account of the circumstances that support or challenge facial PPE use and to inform occupational protection strategies that are responsive to the practical needs and working conditions of CSSD personnel.

## 2 Methods

### 2.1 Researcher qualifications and reflexivity

The research team comprised female researchers with expertise in psychology, nursing, and sterile processing. The semi-structured interviews were conducted primarily by Huiling Rao, who holds a master’s degree in medicine, has received formal training in qualitative research methods, and has published qualitative research. Huiling Rao worked in the same department as the participants and was primarily responsible for research-related activities. She did not hold a direct supervisory, performance-evaluation, quality-control, or managerial role over the participants. She directly approached eligible personnel, provided study information, and obtained their willingness to participate.

Aliya, a sterile processing professional with a bachelor’s degree in nursing and 15 years of relevant experience, contributed primarily to data organization, transcript verification, and thematic analysis. She worked in the same department and had collegial relationships with some participants but held no supervisory or managerial position.

Xue Zhou, a sterile processing professional with 25 years of experience, was responsible primarily for transcribing the interviews, verifying the transcripts, and contributing to the thematic analysis. She also worked in the same department and had collegial relationships with some participants.

Xiaomei Ren, who holds a PhD in nursing and has published research in psychology, was the departmental leader with direct managerial responsibility for the participating personnel. Her role in this study was limited to contributing to refinement of the interview guide and overseeing the overall progress of the project. She was not involved in approaching or recruiting potential participants, obtaining informed consent, conducting interviews, or being present during the interviews. She did not have access to audio recordings or other materials containing participants’ direct identifying information. Her involvement in the later stages of the study was limited to reviewing de-identified interview transcripts, contributing to interpretation and discussion of the emerging findings, and supervising the overall research process. Participants were informed before participation that the departmental leader would not be involved in recruitment or interviewing and would only have access to de-identified transcribed data during the subsequent research process.

Before the study commenced, all members of the research team received training in qualitative research procedures to ensure consistency and methodological rigor throughout data collection and analysis.

### 2.2 Participants and sampling

From January to February 2026, purposive sampling was used to recruit healthcare personnel working in the CSSD of a Grade A tertiary hospital in Chongqing, China. A total of 53 personnel met the eligibility criteria during the study period. From this eligible pool, participants were purposively approached to capture variation in primary work area, occupational role, and length of CSSD work experience, thereby obtaining perspectives from personnel with differing routine tasks and professional backgrounds. Huiling Rao directly approached the selected eligible personnel, provided information about the study, and invited them to participate. Recruitment and analysis proceeded concurrently and ceased after two consecutive interviews generated no new relevant codes, categories, or substantively different thematic information. A total of 20 personnel were approached; all agreed to participate and completed the interview, with no refusals or withdrawals.

The inclusion criteria were as follows: ① having worked in the CSSD for at least 1 year; ② routinely using facial protective equipment during daily work, including medical masks, medical protective goggles, and face shields; ③ being fully conscious, able to communicate clearly, and capable of providing a comprehensive account of their experiences with different types of facial protective equipment; and ④ understanding the purpose of the study, voluntarily agreeing to participate, and providing written informed consent.

The exclusion criteria were as follows: ① personnel undertaking temporary training placements, internships, or temporary assignments in the CSSD; ② personnel working in administrative, managerial, or other non-frontline operational positions; ③ personnel who declined to participate or were unable to complete the interview.

A total of 20 participants were interviewed, including the two participants who took part in the pilot interviews described below. The final two interviews generated no new relevant codes or categories and no substantively different thematic information. Following discussion within the research team, data saturation was considered to have been reached and recruitment was discontinued. No additional interviews were conducted thereafter. All 20 interviews, including the two pilot interviews and the final two interviews used to confirm saturation, were retained in the final analysis, and no interviews were excluded. Participants were assigned identification codes P1–P20. Their demographic and professional characteristics are presented in Table 1.

**Table 1:** Characteristics of the participants (*N* = 20).

Characteristic	Category	*n*	%
Sex	Male	1	5
	Female	19	95
Age, years	20–29	7	35
	30–39	10	50
	≥40	3	15
Primary work area	Decontamination area	8	40
	Inspection, packaging, and sterilization area	7	35
	Sterile storage area	5	25
CSSD work experience, years	1–5	9	45
	6–10	6	30
	>10	5	25
Educational attainment	College diploma or lower	11	55
	Bachelor’s degree or higher	9	45
Occupational role	Nurse	10	50
	Sterile processing technician	10	50

### 2.3 Development of the interview guide

The initial semi-structured interview guide was developed with reference to previous research on facial PPE adherence, including the 2024 version of the Facial Protection Questionnaire (FPQ) reported by King et al. (4), a copy of which was obtained directly from the corresponding author, Prof. Kathryn Nichol. The FPQ was not administered or scored as a quantitative instrument in the present study. It was used to identify potentially relevant areas for exploration and to ensure that the interview guide addressed issues previously associated with facial PPE use. These areas included perceptions of facial protection, protective practices and occupational experiences, organizational support, training, supervision, and recommendations for improvement. The interview guide was adapted to reflect CSSD workflows and common situations involving facial PPE use. The FPQ and previous literature were used only to inform the content of the interview guide and did not provide a predefined coding framework, analytic categories, or themes for the qualitative analysis.

The researchers recognized that their professional backgrounds and familiarity with sterile processing, occupational protection, quality management, and departmental practice could shape their prior assumptions during development of the interview guide. The research team therefore invited experts from different disciplinary backgrounds, including nursing, infection prevention and control, and sterile processing, to review the content of the guide, which was subsequently revised according to their feedback. Questions were designed to be open-ended and addressed participants’ perceptions of facial protection, actual protective practices, difficulties encountered during use, occupational experiences, organizational support, and recommendations for improvement. During the interviews, these questions were used flexibly, and participant-led probing was employed to explore experiences and issues that emerged beyond the topics initially included in the guide.

Before formal data collection, pilot interviews were conducted with two CSSD personnel who met the inclusion criteria. Based on the pilot interviews, minor revisions were made to the wording of selected questions to improve clarity and better reflect terminology commonly used by CSSD personnel in routine work. These revisions involved phrasing only; no substantive interview domain, topic, or intended meaning was added, removed, or changed. Therefore, the pilot and final interview guides addressed the same substantive content. Both pilot participants met the eligibility criteria, and the resulting interview data were comparable in scope and content with those collected subsequently. The two pilot interviews were therefore retained in the final analysis and were included within the total sample of 20 participants (P1–P20). The final interview guide is presented in Table 2.

**Table 2:** Semi-structured interview guide.

Domain	Guiding questions
Perceptions and attitudes toward facial protection	What types of facial PPE do you most commonly use in your daily work, such as surgical masks, N95 respirators, protective goggles, or face shields? How do you understand the importance and protective purposes of wearing facial PPE during routine CSSD work? Are there clear institutional policies, departmental requirements, or training programmes concerning facial protection in your workplace?
Actual use and factors influencing protective decisions	Under what circumstances do you choose to wear a surgical mask, an N95 respirator, protective goggles, or a face shield? How do the work area, task characteristics, or perceived exposure risk influence your choice of facial PPE? Have you experienced shortages of masks, respirators, goggles, or face shields, and how were these shortages managed? How do coworkers and supervisors respond to the implementation of facial protection requirements? What factors do you believe influence facial PPE use in the current healthcare environment, such as policies, workload, workflow, risk perception, or workplace relationships?
Barriers and difficulties during use	Have you experienced discomfort or work-related interference while wearing masks, goggles, or face shields, such as breathing difficulty, fogging, skin problems, restricted vision, or communication barriers? How have these problems affected your work efficiency, task performance, or communication with coworkers? Have you adopted any personal strategies or modifications to reduce these difficulties?
Psychological and occupational experiences	Do you experience psychological burden, anxiety, or emotional discomfort when using facial PPE? Are you concerned that inadequate protection may increase the risk of occupational exposure or transmission to coworkers? How do you balance protective requirements with communication, operational efficiency, and timely completion of instrument reprocessing tasks?
Organizational support and recommendations for improvement	How well does your hospital or department support the supply, allocation, training, and supervision of facial PPE? Which areas require improvement? What changes would you most like to see in equipment availability, product fit and design, training methods, policy implementation, working conditions, or workflow arrangements? How could sustained and correct facial PPE use be better promoted in routine CSSD work?
Additional experiences	Is there anything else you would like to add regarding your experiences, concerns, or recommendations related to facial PPE use?

### 2.4 Interview procedures and transcription

The interviews were conducted in the departmental staff lounge, which provided a quiet and familiar environment for the participants. Before each interview, the interviewer explained the purpose and significance of the study, the interview procedures, the measures used to protect anonymity and confidentiality, and the participants’ right to participate voluntarily and withdraw at any time. Each interview commenced only after both written and verbal informed consent had been obtained. The interviewer avoided introducing personal interpretations, expectations, or assumptions into the questioning process.

Each interview lasted approximately 30–45 min and was audio-recorded in full. Immediately after each interview, the audio recording was transcribed using the speech-to-text function of Baidu Cloud. The interviewer then checked the preliminary transcript against the original audio recording and promptly corrected any transcription errors or inaccuracies to ensure the completeness and accuracy of the data.

Baidu Cloud was used solely for speech-to-text transcription. According to the platform’s privacy statement, user-uploaded content is protected and is not made publicly available. This transcription procedure and the handling of interview data were covered by the ethics approval and informed consent process.

All interviews were conducted in Chinese, and transcription, coding, and analysis were performed in Chinese. Quotations selected for publication were translated into English and checked against the original Chinese transcripts for accuracy.

### 2.5 Data analysis

Data were analyzed using an inductive thematic approach consistent with the qualitative descriptive design. No formal theoretical model, *a priori* coding framework, or predefined analytic categories were imposed on the data. Previous research and the FPQ informed the development of the interview guide, but they were not used as a deductive coding framework during analysis. Coding and theme development were driven primarily by participants’ accounts.

After each interview, two researchers independently read the transcript repeatedly to become familiar with the data and obtain an overall understanding of the participant’s account. Relevant segments of text were assigned initial descriptive codes based on their content and meaning. Codes were compared across interviews, and conceptually related codes were grouped into broader descriptive categories. Similarities, differences, and relationships among these categories were then examined to develop candidate themes. During analysis, emerging interpretations were repeatedly checked against the original transcripts. Differences in coding or interpretation were discussed within the research team, and particular attention was given to findings that extended beyond the anticipated topics and to accounts that differed from dominant patterns.

Saturation was assessed throughout this concurrent process. After each interview, the two researchers compared newly identified codes, categories, and emerging thematic patterns with those identified in previous interviews. When two consecutive interviews produced no new relevant codes or categories and no substantively different thematic information, this assessment was reported to Huiling Rao and Xiaomei Ren and discussed within the research team. After discussion within the research team, data saturation was considered to have been reached, and recruitment was discontinued after the 20th interview. No further interviews were conducted.

The candidate themes were subsequently reviewed against the coded extracts and the complete dataset. Theme boundaries and definitions were refined through iterative discussion within the research team. Particular attention was given to findings that extended beyond the topics explicitly addressed in the interview guide, as well as accounts that differed from dominant patterns across participants. The final themes were agreed upon through team discussion and reflected recurring patterns across participants’ experiences and practices.

To support the credibility of the findings, the preliminary findings, including the themes and subthemes, were returned to five participants (P1, P5, P10, P15, and P20) for participant validation. They were asked whether the findings adequately reflected their experiences and whether any important information had been omitted or misinterpreted. None identified substantive omissions or misinterpretations, and no changes to the thematic structure were required.

NVivo 12 was used to organize transcripts, codes, categories, and themes and to facilitate systematic management of the qualitative data. When differences in coding or interpretation occurred, the researchers discussed the relevant transcript excerpts and analytic rationale until consensus was reached.

The research team recognized that existing workplace relationships and differences in organizational authority could influence participants’ willingness to discuss sensitive topics, including non-adherence, mandatory PPE requirements, supervision, peer reminders, and workplace hierarchy. Particular attention was therefore given to the potential effects of social desirability and perceived power relationships throughout data collection and interpretation. Before each interview, participants were informed that participation was voluntary, that they could withdraw at any time without adverse consequences for their employment or related rights, and that their responses would be anonymized and treated confidentially. The researchers also recognized that their professional experience in sterile processing, occupational protection, quality management, and departmental administration could shape the interpretation of participants’ accounts. During analysis and team discussions, emerging interpretations were repeatedly checked against the original transcripts, and the researchers considered whether professional experience, workplace roles, or prior assumptions might have influenced the developing analysis.

### 2.6 Human rights protection and medical ethics

The study was approved by the Ethics Committee of the First Affiliated Hospital of Army Medical University [approval no. (A) 2025KY219] and was conducted in accordance with the Declaration of Helsinki and relevant principles of medical research ethics. Before each interview, the researcher explained the study purpose, interview content, intended use of the data, confidentiality procedures, and the voluntary nature of participation. All participants provided written informed consent after receiving sufficient information about the study. Participants were informed that they could withdraw from the study at any time without any adverse consequences for their employment or related rights and interests.

To protect participants’ privacy and confidentiality, all interview data were anonymized, and participants were identified using codes P1–P20. Audio recordings, transcripts, and other study-related materials were stored securely in encrypted files and were accessible only to authorized members of the research team for purposes directly related to the study. The data were used exclusively for research analysis and manuscript preparation and were not used for any unrelated purposes. Owing to the sensitive and potentially identifiable nature of the qualitative data, the original data are not publicly available. Reasonable requests for access may be directed to the corresponding author and will be considered subject to ethical approval, the scope of participants informed consent, and applicable privacy and confidentiality requirements.

## 3 Results

A total of 20 CSSD personnel participated in the study and were assigned identification codes P1–P20. Participant characteristics are presented in Table 1. Analysis of the semi-structured interview data generated six themes and 18 subthemes. The findings described how participants understood the meaning of facial protection, made context-dependent protection decisions, responded to institutional requirements and supervision, experienced equipment-related discomfort and interference, managed environmental and workflow constraints, and negotiated the cognitive and interpersonal factors shaping sustained protective practices.

### 3.1 Perceptions of the purpose of facial protection: personal safety, device and patient safety, and responsibility for wider safety

Participants described the most immediate purpose of facial protection as personal protection from occupational exposure. All 20 participants referred to facial PPE as a barrier against hazards encountered during routine CSSD work. Masks and respirators were described primarily in relation to protection of the nose and mouth from droplets or respiratory exposure, whereas goggles and face shields were described as protecting the eyes and face from splashes of blood, body fluids, contaminated liquids, or chemicals (see Table 3).

**Table 3:** Perceptions of the purposes of facial protection.

Theme	Subtheme	Illustrative quotations
Perceptions of facial protection: personal safety, device and patient safety, and responsibility for others	Facial protection as the first barrier against occupational exposure	“The first priority is self-protection. In healthcare, we may come into contact with blood, body fluids, and secretions, which can result in occupational exposure.” (P1)
		“The face shield prevents splashes from reaching the mucous membranes of the eyes and the skin of the face during the procedure. It protects me.” (P4)
	Perceived bidirectional protection: protecting personnel and limiting contamination of medical devices	“In the inspection and packaging area, a surgical mask helps protect the instruments from droplets. In the decontamination area, a face shield or goggles protect us when contaminated liquid splashes.” (P8)
		“In the decontamination area, facial protection is used to protect the operator. In the inspection and packaging area, wearing a mask prevents secondary contamination of the instruments.” (P15)
	Perceived responsibility for protecting others and wider safety	“If you are unwell, you should put on a mask promptly to avoid passing the infection to other people.” (P7)
		“During the pandemic, the department encouraged us to wear N95 respirators because they protected our safety and our families’ safety.” (P9)

Participants’ understanding of protection was not limited to their own safety. Nine participants described facial PPE as providing bidirectional protection. In decontamination-related work, they viewed goggles, face shields, and masks as protecting personnel from exposure to contaminated devices and materials. During inspection, assembly, and packaging, participants perceived surgical masks as helping to limit contamination of cleaned or prepared instruments by workers’ respiratory droplets. Protection was therefore understood by participants as operating in both directions: from contaminated devices to the worker and from the worker to the device.

Twelve participants extended the meaning of facial protection beyond the immediate worker–device interaction. They discussed the risk of transmitting infection to coworkers, patients, or family members. This concern was more prominent when a worker had respiratory symptoms or during infectious disease outbreaks. For these participants, the use of facial PPE also reflected consideration for people who might be affected by workplace exposure or subsequent transmission.

### 3.2 Factors shaping reported protective decision-making: work context, comfort, and unexpected events

Participants adjusted the type and intensity of facial protection according to their assessment of occupational risk. Work area, task characteristics, equipment comfort, and the presence of an infectious disease threat all influenced these decisions (see Table 4).

**Table 4:** Factors shaping reported protective decision-making.

Theme	Subtheme	Illustrative quotations
Factors shaping reported protective decision-making: work context, comfort, and unexpected events	Reported use of enhanced facial protection in perceived high-risk areas	“In the collection area, caps, masks, gloves, face shields or goggles are mandatory.” (P1)
		“In the decontamination area, goggles, a face shield, and a mask are prepared for the workers.” (P15)
	Basic protection and flexible rules in lower-risk areas	“There is no compulsory requirement in my area. People can choose whether to wear a mask.” (P3)
		“A surgical mask is required in the decontamination area. In the packaging area, people may choose whether to wear one.” (P13)
	Reported escalation from surgical masks to N95 respirators during infectious disease threats	“During the most severe phase of the outbreak, everyone was required to wear an N95 respirator, regardless of the work area.” (P1)
		“When there is no airborne or other high-risk disease, we use surgical masks. During the 2022 outbreak, we used N95 respirators because they provided a higher level of protection.” (P9)

Sixteen participants described decontamination and instrument collection as high-risk activities. These tasks involved direct contact with contaminated medical devices and the possibility of exposure to blood, body fluids, splashes, and aerosols. Several participants referred to face shields, goggles, and masks as mandatory equipment in decontamination or collection areas.

All participants distinguished routine protection in inspection, packaging, sterile storage, or distribution areas from the enhanced protection used during decontamination. Surgical masks were the main form of facial protection in these settings. Goggles and face shields were seldom used unless a specific task created a splash or aerosol risk. Workers sometimes adjusted their practices in response to heat, humidity, respiratory discomfort, or their assessment of the immediate task. This flexibility reflected a combination of institutional rules, perceived risk, and personal tolerance.

All 20 participants described a shift toward higher-level protection during the COVID-19 pandemic or when respiratory infectious diseases were suspected. N95 respirators replaced surgical masks in many situations.

### 3.3 Participants perceived policies, training, and supervision as supporting expected facial protection practices

Participants described facial protection as a practice shaped by organizational arrangements as well as personal awareness. Written standards defined the minimum requirements for different work activities. Participants reported that training helped them understand these requirements and how they should be applied in daily work, while supervision and immediate feedback served as reminders of expected protective practices (see Table 5).

**Table 5:** Participants’ perceptions of organizational support for expected facial protection practices.

Theme	Subtheme	Illustrative quotations
Participants perceived policies, training, and supervision as supporting expected facial protection practices	Professional and institutional standards provided explicit requirements for protective practices	“The 2016 CSSD standard clearly specifies the protective requirements for each work area. Our department also has a separate occupational protection policy.” (P1)
		“The requirements are displayed on the wall near the entrance to the work area, with both pictures and written instructions.” (P12)
	Multilevel training supported understanding of protective requirements	“Protective requirements are included in our routine learning. A designated person leads the session, and questions are asked afterwards to check whether staff have understood it.” (P1)
		“The hospital provided training before I started work. I received another session when I entered the department, followed by regular training afterwards.” (P12)
	Team-leader supervision and peer feedback reinforced adherence	“Facial protection is included in our second- and third-level quality-control inspections, and we check it directly in the work area.” (P1)
		“When we see someone working without a mask, we remind them to put it on immediately.” (P2)
		“If I see a coworker using an air gun without a mask, I ask them to put it on because the procedure may generate aerosols.” (P16)

Eighteen participants referred to professional standards, hospital policies, departmental procedures, or written operating requirements. These documents specified the facial PPE required for particular work areas and tasks. Participants described these standards as reducing uncertainty about what facial PPE was expected for particular work areas and tasks. Written instructions, photographs, and notices displayed near entrances or changing areas served as visible reminders before personnel entered the work area.

All 20 participants described receiving facial protection training at one or more organizational levels. New staff training focused on basic requirements and recommended protective practices. Routine team or departmental sessions reinforced the purpose of facial PPE and reviewed relevant standards. Some participants described short learning sessions, periodic lectures, written manuals, questioning, or practical instruction.

Eighteen participants discussed supervision, inspection, or reminders from team leaders and coworkers. Formal supervision included quality-control rounds, on-site checks, and random inspections. Participants described these activities as opportunities for managers or team leaders to identify practices perceived to be inconsistent with departmental requirements and to provide immediate reminders. Peer feedback was described as a more immediate form of reminder during daily work. Participants described reminding coworkers to wear a mask when required or to adjust facial PPE when they perceived the level of protection to be insufficient for the task.

### 3.4 Reported discomfort and work-related interference associated with facial PPE

Negative experiences with facial PPE were common across the interviews. Participants described device-specific problems, including pressure and skin discomfort associated with masks, goggles, and face shields; fogging or visual obstruction associated mainly with goggles and face shields; and communication difficulties while wearing masks, respirators, or face shields (see Table 6).

**Table 6:** Discomfort and work-related interference associated with facial protective equipment.

Theme	Subtheme	Illustrative quotations
Reported discomfort and work-related interference associated with facial PPE	Pressure, skin discomfort, and irritation during facial PPE use	“If the goggles are loose, they fog up. If they are tight, the area around my eyes hurts. The elastic straps also make my ears hurt.” (P7)
		“If the face shield makes my face itch, I keep wanting to adjust it, and that can affect my efficiency.” (P13)
	Fogging and visual obstruction during goggles and face-shield use	“The goggles sometimes fog up. If the face shield is splashed with contaminated water, I cannot see clearly.” (P7)
		“Fogging definitely affects visibility. We usually remove the face shield and replace it with a new one.” (P19)
	Masks, respirators, and face shields were associated with communication difficulties	“Wearing a mask definitely affects communication efficiency.” (P12)
		“The equipment in the work area is noisy. When we wear masks, people may not understand immediately, so we have to raise our voices or repeat the message.” (P1)
	Poor fit created additional problems	“The hospital purchases one brand, and the mask sizes are fixed. They do not fit every facial shape or body size.” (P3)
		“For people with fuller faces, the mask feels tight. For thinner people, it is loose, so they have to tie knots in the straps.” (P20)

Eighteen participants described at least one form of physical discomfort associated with masks, goggles, or face shields. Participants described a trade-off between perceived protection and comfort: tighter-fitting equipment was perceived as more secure but increased pressure and pain, whereas loosening straps relieved discomfort but was perceived to reduce stability or sealing. Participants also mentioned facial marks, dizziness, itching, allergic reactions, and skin irritation. The discomfort accumulated with wearing time. A tighter fit provided better protection but increased pressure and pain. Loosening the straps reduced discomfort while weakening stability or facial sealing. Participants sometimes adjusted straps, added clips or padding, or temporarily left the work area to relieve the pressure. All participants reported fogging or visual obstruction when using goggles or face shields, which they perceived as interfering with task performance and operational efficiency. Fogging was described in relation to breathing, heat, humidity, and prolonged wear. Participants also noted that reusable face shields could become blurred after repeated cleaning or disinfection. These visual problems were perceived to interfere with task performance and operational efficiency.

Eighteen participants described difficulty hearing or being heard while wearing masks, respirators, or face shields. The problem was more apparent in the decontamination area, where washers and other equipment generated continuous noise. Participants responded by speaking more loudly, repeating information, moving closer to coworkers, or temporarily adjusting their PPE.

Sixteen participants reported that standard-sized facial PPE did not accommodate differences in facial shape and size. A mask could be tight for one worker and loose for another. Poor fit led to pressure, ear pain, slippage, air leakage, reduced sealing, and the need for repeated adjustment. Participants frequently modified the equipment themselves. They stretched ear loops, tied knots, wrapped straps several times, added clips, or placed padding beneath pressure points.

### 3.5 Physical working conditions and workflow shaped participants’ experiences of sustained facial PPE use

Participants described the physical work environment and organization of CSSD tasks as important conditions shaping their ability and willingness to tolerate facial PPE during prolonged work (see Table 7).

**Table 7:** Participants’ experiences of physical working conditions and workflow during sustained facial PPE use.

Theme	Subtheme	Illustrative quotations
Physical working conditions and workflow shaped participants’ experiences of sustained facial PPE use	Heat and inadequate ventilation intensified discomfort	“When the workload is heavy and the environment is humid and hot, I become irritable. Wearing protective equipment at that time makes me feel as though I cannot breathe.” (P12)
		“The weather is hot and the air feels stuffy. Wearing a surgical mask and face shield makes the discomfort worse.” (P11)
		“When the air conditioning failed, the mask became so wet that water was dripping from it.” (P15)
	Production-line workflow and prolonged continuous work challenged tolerance of facial PPE	“After wearing the protective equipment for several hours, I want to take it off, relax, and get some air. This is more obvious in the decontamination area.” (P1)
		“Workers may remain in the same area for three or four hours. We need to consider how long they can tolerate the equipment.” (P15)
		“During high-intensity work, there should be a short break of several minutes between continuous periods of work.” (P15)

Thirteen participants described heat, humidity, or poor ventilation as factors that made facial PPE more difficult to tolerate. These conditions were commonly reported in decontamination areas, where cleaning equipment, waterproof protective clothing, and enclosed or negative-pressure environments increased the sensation of heat and moisture. Participants described sweating, damp masks, breathlessness, irritability, and a strong need to leave the work area temporarily. Wearing a mask or face shield became more burdensome when room temperature and ventilation were poorly controlled.

Eight participants described CSSD work as repetitive, continuous, and closely paced. Personnel often remained in the same work area for several hours while completing instrument collection, cleaning, inspection, or packaging tasks. PPE-related discomfort accumulated during these uninterrupted periods. Some participants reported wanting to remove or loosen their PPE after several hours. Short breaks for drinking water, using the toilet, or breathing outside the work area provided temporary relief.

### 3.6 Complex influences on reported facial PPE practices: differences in understanding, interpersonal dynamics, and mandatory requirements

Participants described facial PPE use as a negotiated practice. Differences in professional background, work role, length of experience, and personal risk perception shaped how facial protection requirements were understood and interpreted. Coworker and supervisor reminders were described as influencing reported protective practices, although participants indicated that their reception depended on interpersonal relationships and workplace hierarchy (see Table 8).

**Table 8:** Complex influences on reported facial PPE practices.

Theme	Subtheme	Illustrative quotations
Complex influences on reported facial PPE practices: differences in understanding, interpersonal dynamics, and mandatory requirements	Understanding of the need for facial protection varied by role, experience, and professional background	“New staff do not know the work yet, so they follow what they are told. Some experienced staff know that they should wear the equipment, but they still decide according to their own judgement.” (P1)
		“The quality-control staff are nurses, while some of the personnel being reminded have worked here for many years. Seniority can affect how they respond to the reminder.” (P18)
	Peer reminders were shaped by interpersonal relationships and workplace hierarchy	“Quality-control staff may be reluctant to offend people. If occupational protection is mentioned repeatedly, some workers may become resistant.” (P15)
		“Some people are willing to accept a reminder because they see it as being for their own good. Others feel that you are finding fault with them.” (P18)
	Mandatory requirements produced passive acceptance and endurance	“Everyone knows that it is important, but the equipment is uncomfortable and we still have to wear it.” (P20)
		“There is no other solution. If it is required, you still have to wear it.” (P11)

Eleven participants described differences in protective awareness among staff groups. They perceived new employees as tending to follow instructions closely while learning departmental expectations. Experienced personnel were described as having greater familiarity with occupational risks, although some participants felt that familiarity with routine work could also lead workers to rely more heavily on personal judgement. Participants reported that some experienced workers modified their protective practices when they perceived the risk to be low or when facial PPE interfered with comfort or efficiency.

Eight participants described reminders as a sensitive form of workplace interaction. A reminder could be understood as care and mutual protection when it came from a trusted coworker. The same message could be perceived as criticism when it was repeated, delivered by a less senior colleague, or associated with formal quality control. Participants perceived team leaders and quality-control personnel as having greater authority to address practices that did not align with departmental requirements. Their role also placed them in a difficult interpersonal position. Some participants worried that frequent reminders might offend coworkers or create the impression that they were finding fault.

Twelve participants described situations in which they continued to wear facial PPE despite discomfort or reluctance because the rules required it. This response was most apparent in decontamination areas, during high-risk procedures, and during infectious disease outbreaks. Participants accepted that the equipment had to remain in place, even when it caused pressure, heat, breathing discomfort, or emotional resistance.

## 4 Discussion

This study explored CSSD personnel’s experiences of using facial PPE during routine hospital operations. Participants recognized facial protection as a safeguard for their own health, the cleanliness of reprocessed medical devices, patient safety, and the safety of people around them. Their protective decisions remained strongly dependent on the perceived risk of the task, the work area, equipment comfort, and the presence of an infectious disease threat. Policies, multilevel training, supervision, and peer reminders were perceived as supporting expected practices, yet sustained use was challenged by pressure-related discomfort, poor fit, fogging, communication difficulties, heat, inadequate ventilation, and prolonged continuous work. Reported facial PPE practices also reflected differences in professional experience, individual risk interpretation, workplace relationships, and responses to mandatory requirements. These findings suggest that facial PPE use in CSSDs should not be understood solely as an issue of individual knowledge or willingness. It is shaped by the interaction between equipment design, work processes, physical conditions, organizational arrangements, and interpersonal dynamics.

Participants in this study viewed facial PPE as protection for themselves, their coworkers, medical devices, and patients. CSSD personnel routinely handle instruments contaminated with blood, body fluids, and other organic material. Manual cleaning, brushing, flushing, and rinsing may generate droplets and splashes that reach the worker’s face (13). Ofstead et al. (3) found that visible droplets were produced during most simulated instrument-reprocessing activities and could travel up to 5 feet from the sink, supporting participants’ perception that masks, goggles, and face shields form an essential barrier against occupational exposure. This protective role is similar to that of facial PPE in direct patient care, where healthcare workers use masks and eye protection when contact with respiratory secretions, blood, body fluids, or aerosols is anticipated (14). A systematic review by Chu et al. also found that face masks and eye protection were associated with a lower risk of coronavirus transmission in healthcare and community settings (15). Surgical masks may also contribute to source control when workers have respiratory infections by reducing the release of respiratory viruses in droplets or aerosols (16). During inspection and packaging, workers speak, breathe, and work close to devices that have completed cleaning and disinfection but have not yet been sterilized. Participants believed that wearing a mask reduced the possibility of respiratory droplets contaminating these instruments. Philips et al. reported that speaking without a mask produced bacterial contamination on nearby culture plates, while a fresh surgical mask almost completely prevented bacterial dispersal from the upper airway (17). Microbial contamination has also been detected on a proportion of surgical instruments sampled in CSSD areas before sterilization, including after cleaning, disinfection, and packaging, indicating that recontamination can occur during the reprocessing pathway (18). Such contamination does not directly mean that a validated sterilization cycle will fail, but it increases the bioburden entering sterilization (18, 19). Therefore, facial PPE throughout the CSSD device reprocessing pathway is an important measure for protecting personnel, coworkers, and patients.

Participants in this study identified clear institutional requirements, multilevel training, workplace supervision, peer reminders, and reliable access to facial PPE as factors perceived as supporting expected protective practices. These findings are consistent with previous research indicating that unit-level safety climate, communication, and organizational support are associated with self-reported adherence to facial protective equipment (20). Harrod et al. found that unclear protocols and different interpretations of clean and contaminated spaces could lead to inconsistent PPE use, while education based on real work situations helped personnel apply protective requirements more effectively (21). Quantitative evidence also supports the role of training and supervision. Bekele et al. reported that healthcare workers who had received infection prevention training were more likely to comply with standard precautions, and supportive supervision showed an even stronger association with compliance (22). A multisite study of home-care workers found that better knowledge, convenient access to facial protective equipment, and stronger organizational support for occupational health and safety were associated with higher self-reported adherence (4). Peer influence also appears to be important. Tsang et al. identified professional responsibility, social influence, constructive feedback, regular audits, and PPE champions as potential facilitators of appropriate PPE use (23). The present study adds a CSSD perspective to this evidence. Participants described a complete system in which professional standards defined expected practices, hospital and departmental training translated these requirements into routine procedures, and team leaders or coworkers provided reminders when practices were perceived to be inconsistent with departmental requirements. In this setting, the main challenge was less about obtaining PPE and more about maintaining protective practices in accordance with departmental requirements during repetitive tasks and routine operations.

Participants identified several objective barriers to sustained facial PPE use, including physical discomfort, poor fit, fogging, communication difficulties, heat, and prolonged continuous work. CSSD personnel often remain in enclosed, hot, humid, and noisy work areas for several hours while completing repetitive and closely connected reprocessing tasks. These working conditions may intensify discomfort and make facial PPE more difficult to tolerate over time. PPE-associated headaches and discomfort during prolonged use have also been reported among healthcare workers in other settings (24). King et al. (25) found that discomfort, impaired visibility, and communication difficulties were more frequently reported by workers with lower adherence to facial protective equipment. Harrod et al. (21) also showed that PPE use was influenced by equipment accessibility, organizational processes, perceived risk, and the practical demands of clinical work. Physiological and survey-based studies have documented sweating, headache, dizziness, breathing discomfort, fatigue, and reduced task performance among healthcare workers wearing PPE, particularly during prolonged work in warm environments (26, 27). Equipment design may further increase these difficulties. A human factors study of face shields identified adjustable tension, shifting load bearing away from the temples, anti-fogging, ventilation, freedom of movement, and durability as important design elements for usability (28). These findings are consistent with participants’ descriptions of facial pressure, skin irritation, fogging, heat, and the need to modify masks or straps to accommodate different facial shapes.

Subjective barriers to sustained facial PPE use were related to risk appraisal, personal experience, workplace relationships, and acceptance of mandatory requirements. Previous studies have shown that knowledge of infection-control principles and concern about transmitting infection can promote PPE adherence (5). Risk perception also influences whether workers consider a protective measure necessary in a specific situation. Fix et al. (29) found that healthcare workers sometimes relied on their clinical experience rather than formal protocols when judging the need for respiratory protection. These findings are reflected in the present study, where participants applied stricter protection in decontamination areas and during infectious disease outbreaks but adopted more flexible practices in areas perceived as cleaner or lower risk. CSSD personnel have limited direct contact with patients, and the consequences of inadequate facial protection may be less immediately visible than those encountered in clinical wards. Familiarity with routine tasks may further encourage experienced workers to substitute personal judgement for standardized requirements. Workplace relationships added another layer to this process. Monitoring by supervisors can improve adherence, while coworkers’ non-compliance can weaken accepted safety norms (30). Participants in this study described peer reminders as sensitive interactions shaped by seniority, familiarity, and the authority of the person giving the reminder. Advice intended as mutual protection could be interpreted as criticism or fault-finding.

### 4.1 Practice considerations for facial PPE use in CSSDs

Participants’ accounts, considered alongside the wider literature, suggest several practice considerations for supporting appropriate facial PPE use in CSSDs. Clear departmental protocols aligned with relevant professional standards may help clarify the facial PPE expected for different work areas and tasks, including the handling of contaminated instruments and activities such as manual cleaning, rinsing, drying, inspection, and packaging. Enhanced protection may be particularly important for procedures involving splashes, aerosol generation, or exposure to highly infectious materials, while unnecessarily high levels of protection may increase discomfort without providing additional occupational benefit.

Participants’ accounts particularly highlighted the importance of fit, comfort, and ease of use when selecting facial PPE. Providing masks, respirators, goggles, and face shields in a range of sizes and designs, and allowing staff to trial products before procurement where feasible, may improve equipment acceptability and tolerance. Lightweight materials, adjustable straps, effective anti-fogging properties, adequate ventilation, and designs that minimize pressure on the ears, nasal bridge, and forehead may further improve tolerance during prolonged use. Routine mechanisms for staff feedback could also help identify problems such as lens fogging, skin irritation, inadequate sealing, slippage, or poor fit and inform subsequent equipment selection or adjustment.

Consistent with previous evidence, structured and task-specific training may further support appropriate facial PPE practices. Training for newly employed personnel could address the selection, donning, use, removal, and disposal or reprocessing of facial PPE before independent work, with subsequent theoretical and practical sessions used to reinforce protective requirements in routine practice.

Participants’ descriptions of heat, humidity, prolonged work, and accumulated discomfort suggest that attention to working conditions and workflow design may help support appropriate and sustained facial PPE use. Regular attention to temperature, humidity, ventilation, and noise may help identify environmental conditions that increase PPE-related discomfort. Where feasible, adjustments to air-conditioning, dehumidification, staffing, task allocation, and work schedules could reduce prolonged uninterrupted PPE use and provide opportunities for safe PPE replacement, hydration, and short periods of recovery. Staff rotation and scheduled short breaks may also reduce discomfort and fatigue during prolonged work. Supportive supervision and constructive feedback may help reinforce expected protective practices without relying primarily on criticism or punitive measures.

### 4.2 Limitations and future directions

This study has several limitations. Participants were recruited from the CSSD of a single tertiary hospital in Chongqing, China. Staffing arrangements, protective policies, equipment supply, and working conditions may differ across hospitals and regions, which limits the transferability of the findings. Future studies should include CSSD personnel from hospitals of different levels, geographical regions, and organizational models to examine whether similar experiences occur in other settings.

This study did not directly observe the duration, correctness, or consistency of facial PPE use. Larger mixed-methods studies could develop and test quantitative instruments based on the themes identified here and examine the relationships among risk perception, equipment comfort, working conditions, organizational support, and PPE adherence.

The study examined perceived links between facial PPE use, instrument cleanliness, and patient safety but did not measure microbial contamination, occupational exposure incidents, sterilization outcomes, or healthcare-associated infections. Laboratory and observational studies are needed to assess whether mask use during inspection and packaging reduces contamination of cleaned instruments before sterilization.

All members of the research team worked in the same department as the participants, and one researcher had direct managerial responsibility for the participating personnel. The departmental leader did not directly recruit or interview participants, but existing workplace relationships and perceived organizational hierarchy may still have influenced participants’ willingness to disclose sensitive experiences. Non-adherence or dissatisfaction may have been underreported, whereas descriptions of training, supervision, organizational support, and compliance with expected practices may have been presented more favorably.

Because the researchers and participants worked in the same department, participants were already familiar with the researchers’ professional roles before recruitment. Before participation, they were also informed of the researchers’ respective roles in the study. Some participants also recalled facial PPE practices during the COVID-19 pandemic, including the escalation to N95 respirators during the 2022 outbreak. Because these experiences were reported retrospectively several years later, they may have been affected by recall bias. Findings concerning past emergency practices should therefore be interpreted as participants’ recollections.

## 5 Conclusion

This study shows that CSSD personnel understand facial PPE as an important measure for protecting themselves, coworkers, medical devices, and patients. Participants reported protective practices reflected perceived task-related risk, institutional requirements, training, supervision, equipment fit, physical working conditions, workflow, and workplace relationships. Clear standards and organizational support were perceived to reinforce expected protective practices, whereas discomfort, fogging, poor fit, heat, prolonged continuous work, low perceived risk, and passive responses to mandatory rules were described as barriers to adherence. These findings suggest that improving facial protection in CSSDs involves more than knowledge-based education or compliance monitoring. Task-based protective requirements, usable and well-fitting equipment, practical training, supportive feedback, improved environmental conditions, and appropriate opportunities for recovery during prolonged work may support appropriate and sustained facial PPE use. A worker-centred and system-based approach may help promote more consistent and internally accepted protective practices.

## Data Availability

The datasets presented in this article are not readily available owing to the sensitive and potentially identifiable nature of the qualitative data, the original data are not publicly available. Reasonable requests for access may be directed to the corresponding author and will be considered subject to ethical approval, the scope of participants informed consent, and applicable privacy and confidentiality requirements. Requests to access the datasets should be directed to rhl1997@tmmu.edu.cn.
